# Study on the biological effects of ZnO nanosheets on EBL cells

**DOI:** 10.3389/fbioe.2022.915749

**Published:** 2022-10-04

**Authors:** Mei Li, Yonghua Ma, Xiaodi Lian, Yan Lu, Yuanyuan Li, Yao Xi, Xiaolin Sun

**Affiliations:** ^1^ College of Veterinary Medicine, Gansu Agricultural University, Lanzhou, China; ^2^ Lanzhou Institute of Biological Products Limited Liability Company, Lanzhou, China; ^3^ Northwest Normal University, Lanzhou, China

**Keywords:** nanomaterials, EBL cells, cytotoxicity, apoptosis, reactive oxygen species

## Abstract

In this study, the biological effects of ZnO nanosheets were initially investigated using embryonic bovine lung (EBL) cells cultured *in vitro* as a model. ZnO nanosheets were prepared by a hydrothermal method, and their structure and morphology were characterized, and their effects on EBL cell viability, oxidative stress, cell proliferation, and apoptosis were investigated. The results showed that 12.5 μg ml^−1^ ZnO nanosheets can cause morphological changes in EBL cells. The toxic effects of ZnO nanosheets on EBL cells were time-dependent. Caspase-3 activity in EBL cells changed under certain conditions with the introduction of 25 μg ml^−1^ ZnO nanomaterials, and EBL cell apoptosis was promoted. Under different concentration and time effects, ZnO nanosheets induced an increase in ROS levels in EBL cells, indicating that they have an oxidative damage effect on cells. The toxic effects of ZnO nanosheets on EBL cells were discussed, including concentration effect, time effect, and cytotoxic effect, which eventually led to cell oxidative damage.

## 1 Introduction

Nanotechnology was first proposed by R. Feynman, a Nobel laureate in physics, in the 1960s, and he predicted that changing or controlling the microstructural arrangement of objects would produce materials with new properties (Bayda et al., 2019). Nanotechnology is a technology that uses a single atom or molecule to make materials (He et al., 2019). Nanomaterials refer to materials with at least one dimension in nanosize (1–100 nm) in three-dimensional space or composed of them as basic units, which is about the scale of 10–1,000 atoms closely arranged together (Liao et al., 2020). Nanomaterials are now widely used in the biomedical field because of their unique structure and properties. Scientists are currently focusing on the synthesis and modification of nanomaterials and their applications in medical imaging, tumor treatment, and so on (Fu et al., 2018; Wu et al., 2019). Due to the good adsorption effect of nanomaterials, drugs or agents can be adsorbed on the surface of nanomaterials and localized to tumor sites to play a certain role in improving drug efficacy, reducing the dosage of drugs, and reducing toxic side effects, etc. (Tiwari et al., 2017; Muniandy et al., 2019). Alexiou et al. (2003) performed regional arterial perfusion in an animal model and found that the anti-cancer drugs were loaded onto the surface of magnetic nanoparticles, and, under the influence of an applied magnetic field, the nanoparticles were able to enter the tumor site with blood circulation and penetrate into the tumor tissue, which greatly improved the therapeutic efficiency of the given drugs. To be better applied in biomedical fields, research studies on the overall effects of nanomaterials on biological organisms, cellular effects, and related molecular mechanisms have also attracted extensive attention. The cell biological effects of nanomaterials mainly include the transmembrane mechanism, intracellular localization and metabolism, cell viability, cell proliferation, and apoptosis. Studies have shown that nanoparticles enter cells through pinocytosis (Ehsani et al., 2022; Abdul Manaf et al., 2021; Liu et al., 2019; Reshma et al., 2017). This process of binding nanomaterials to carriers and receptors on the cell surface causes denaturation of the relevant protein structures and receptors, resulting in blocked nutrient acquisition and cell growth inhibition (Lee et al., 2019). Nanomaterials form many free radicals in cells, which bind to DNA, RNA, and other genetic substances, resulting in erroneous genetic information and cell death (Ma et al., 2021; Hussain, 2016).

ZnO nanomaterials are a new multifunctional inorganic material. In recent years, it has been found that they show many special functions in catalysis, optics, magnetism, mechanics, and so on, which makes them have important application value in many fields such as ceramics, chemical industry, electronics, and optics (Hu et al., 2021; Yilmaz et al., 2021; Singh, 2019; Singh, 2018). In addition, the biological functions of ZnO nanomaterials have attracted the attention of biomaterial scientists and surgeons. The excellent electrical properties of ZnO nanomaterials make them suitable for the preparation of biosensors (Beitollahi et al., 2020). Their excellent catalytic, antibacterial, and biocompatible properties allow ZnO nanomaterials to be used for tissue regeneration, bacterial drug resistance, and trauma dressings (Ellis-Behnke et al., 2006; Zhang et al., 2020). It has been reported that ZnO nanomaterials are among the most promising inorganic antibacterial materials due to their good stability, remarkable antibacterial activity, and low cost (Verma et al., 2018; Li et al., 2021). It is noteworthy that the cell biological effects of ZnO nanomaterials are directly related to their applications in drug development, bioassay, food preservation, and environmental purification, which is a prerequisite for further exploration of *in vivo* conditions (Duan et al., 2021).

Embryonic bovine lung (EBL) cells are a type of primary cells established from fetal cattle lung tissue at about 7 months of gestation, and the cells are an important cell model for studying viral and bacterial diseases in cattle (Sobotta et al., 2017). Although a large number of articles on nanoparticle toxicity have been published in the scientific literature, data on the effects of ZnO nanosheets on EBL cells are still limited. In this study, the ZnO nanosheets were prepared by the hydrothermal method and characterized, and their active effects on EBL cells, oxidative stress response, and effects on cell proliferation and apoptosis were investigated. The results provided data support for the safe application of ZnO nanomaterials in biotechnology and biomedicine and provided a basis for the design of nanoparticles with different biological effects.

## 2 Materials and methods

### 2.1 Cell line

EBL cells were purchased from Shanghai Fusheng Industrial Co.

### 2.2 Main reagents

Zn(CH_3_COO)_2_^.^2H_2_O, ammonia, deionized water, anhydrous ethanol, and fetal bovine serum (FBS) were purchased from Biological Industries; six-well cell culture plates, 96-well cell culture plates, T25 cell culture flasks were purchased from Shanghai Biotech Bioengineering Co.; MTT cell proliferation and cytotoxicity assay kit, Bradford method protein concentration assay kit, caspase-3 activity assay kit, reactive oxygen species assay kit were purchased from Beijing Solarbio Company.

### 2.3 Preparation and characterization of ZnO nanomaterials

For Zno nanomaterial preparation, 1.190 g of Zn(NO_3_)_2_.6H_2_O, 0.100 g of CTAB, 0.100 g of SDS, and 0.500 g of NaOH were weighed, fully dissolved in 30 ml of deionized water, transferred the reaction solution to a 50-ml autoclave, heated to 140°C, and allowed to react for 10 h. The precursor solution was centrifuged five times, dried at 60°C for 4 h, and calcined at 500°C for 2 h in an annealing furnace to obtain the white product, which was stored at 4°C until further use.

The structure and morphology of the prepared ZnO nanomaterials were characterized by XRD, scanning electron microscopy (SEM), and transmission electron microscopy (TEM).

### 2.4 Reagent preparation

For reagent preparation, 10% FBS DMEM (100 ml) was prepared by mixing 10 ml FBS, 89 ml DMEM, and 1 ml antibiotic mixture (penicillin and streptomycin).

Cell lyophilization solution (100 ml) was prepared by mixing 70 ml of 10% FBS DMEM and 20 ml of FBS, and 10 ml of DMSO was slowly dropped in and finally mixed well.

ZnO nanomaterials dispersion: 0.008 g of nanomaterials was weighed in a test tube, 4 ml of DMEM was added, shaken, and mixed on a vortex shaker and then sonicated it to a final concentration of 2 mg ml^−1^. Finally, the samples were diluted with DMEM into different concentrations and stored at 4°C until further use.

### 2.5 Cell culture

After taking out the frozen cells from liquid nitrogen, they were quickly put into a 37°C water bath and shaken gently to completely dissolve them; the dissolved cytoplasm was transferred into the centrifuge tube and centrifuged at 800 r^.^ min^−1^ for 10 min; the supernatant was discarded, and 10% FBS DMEM culture medium was added, gently blown to make it even, inoculated it in the culture bottle, supplied 4 ml of culture medium, and placed it in the incubator (37°C and 5% CO_2_).

### 2.6 Cell morphology observation

EBL cells cultured to the logarithmic phase were dissociated with 0.25% trypsin and inoculated into six-well plates with a concentration of 6 × 10^5^ cells/well. After being cultured in the cell incubator for 24 h, 1 ml of ZnO nanosheet dispersion with different concentrations (12.5, 25, 50, 100, and 200 μg ml^−1^) was added. The control group was added with 1 ml DMEM culture medium, and each group was set with three multiple wells. After 24 h of culture, changes in cell morphology were observed.

### 2.7 Cell viability assay

An MTT assay was used to detect the effects of ZnO nanosheets on cell proliferation. Cells cultured to the logarithmic phase were dissociated with 0.25% trypsin, inoculated in 96-well plates with a concentration of 6,000 cells/well and incubated in a cell culture incubator for 24 h. After discarding the original culture medium, 200 μL of different concentrations of ZnO nanosheet dispersion (12.5, 25, 50, 100, and 200 μg ml^−1^) was added, and six replicate wells of each concentration were set up at the same time. The control group was incubated for 24, 48, and 72 h, respectively. The supernatant was discarded, and 10 μL of MTT solution and 90 μL of DMEM were added to each well. A blank control was set, and after 4 h of incubation, the supernatant was discarded, 110 μL of formazan lysate was added to each well, and the shaker was shaken at low speed for 10 min, and the absorbance value of each well was measured at 490 nm on an enzyme marker. The cell viability was calculated using the following formula: cell viability = (experimental group - blank group)/(control group - blank group) × 100%.

### 2.8 Effects of ZnO nanosheets on apoptosis of EBL cells

#### 2.8.1 The caspase-3 activity reflects apoptosis

Cells cultured to the logarithmic phase were dissociated with 0.25% trypsin, inoculated in six-well plates with a concentration of 1.0 × 10^6^ cells/well and incubated in a cell culture incubator for 24 h. After discarding the original culture medium, 2 ml of ZnO nanosheet dispersion at different concentrations (12.5, 25, 50, 100, and 200 μg ml^−1^) was added, and a control group was set up; three replicate wells were set up for each group and incubated for 2, 4, 8, 16, and 24 h; the original culture medium was discarded, the cells were dissociated with 0.25% trypsin, and the cells were blown with DMEM and transferred to a 1.5-ml centrifuge tube, centrifuged at 1,500 r min^−1^ for 10 min, and the supernatant was discarded. Caspase-3 reagent II was added to the cell precipitate, the cells were resuspended, allowed to stand on ice for 15 min, and centrifuged at 15,000 g/min at 4°C for 15 min; the supernatant was aspirated into a new 1.5-ml EP tube on ice, the reagents required for the analysis were added into the tube, and the blank tube was placed into a 96-well plate according to the system of 100 μL and incubated at 37°C for 60 min, and the absorbance value of each well was measured at 405 nm.

#### 2.8.2 Flow cytometry detection of apoptosis

Cells cultured to the logarithmic phase were dissociated with 0.25% trypsin and inoculated in six-well plates with a concentration of 1.0 ×10^6^ cells/well and incubated in a cell culture incubator for 24 h. Then, 2 ml of ZnO nanosheet dispersion at a concentration of 25 μg ml^−1^ and 200 μg ml^−1^ was added, while a control group was set up and incubated for 24, 48, and 72 h, respectively. After 72 h, the original culture solution was collected into a 1.5-ml EP tube, 0.25% trypsin was used to dissociate the cells, and the cells were blown with DMEM and transferred to a 1.5-ml centrifuge tube and centrifuged at 1,500 r min^−1^ for 10 min; the supernatant was carefully discarded, the cells were resuspended with 1 ml PBS, centrifuged again, and the supernatant was discarded. The cells were resuspended with 1× binding buffer, the concentration was adjusted to 5 × 10^6^, 100 μL of cell suspension was taken in the centrifuge tube, 5 μL annexin was added and mixed well at room temperature and avoided light for 5 min, 10 μL of PI solution and 400 μL PBS were added, and the flow analysis was immediately performed. Blank tubes and single-staining tubes were set up at the same time.

### 2.9 Effects of ZnO nanosheets on reactive oxygen species in EBL cells

Cells cultured to the logarithmic phase were dissociated with 0.25% of trypsin and inoculated into six-well plates with a concentration of 1.0 × 10^6^ cells/well and incubated in a cell culture incubator for 24 h. After discarding the culture medium, 2 ml of ZnO nanosheet dispersion at a concentration of 25 μg ml^−1^ and 100 μg ml^−1^ was added. For the Positive control group, 2 ml of DMEM was added with 2 μL of reactive oxygen positive control reagent (Rosup), and for the negative control group, 2 ml of DMEM was added; three replicate wells were set up for each group. After incubation for 4 and 24 h, the culture medium was discarded, and 1 ml of DCFH-DA application solution (10 μmoL L^−1^) was added to each well; the cells were incubated at 37°C for 20 min; the application solution was discarded, and the cells were washed three times with DMEM and immediately placed in an inverted fluorescence microscope for cell detection.

### 2.10 Statistical analysis

SPSS statistics 25 was used to analyze the significance of differences between sample data, and the data were represented by mean ± standard deviation. *p* > 0.05 denotes that the difference is not significant, and *p* < 0.05 denotes that the difference is significant.

## 3 Results and discussion

### 3.1 Characterization of ZnO nanosheets

**FIGURE 1:**
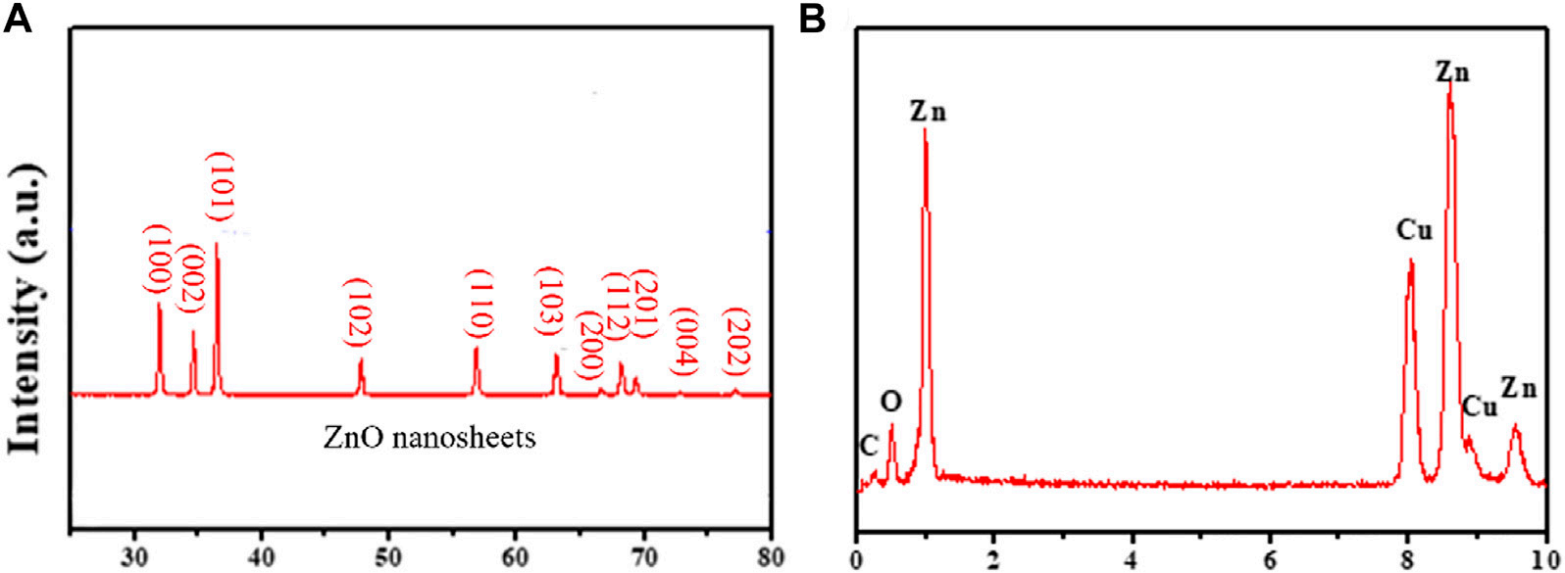
XRD patterns **(A)** and EDX patterns **(B)** of ZnO nanomaterials.

The crystal structure and purity of ZnO nanosheets were characterized using an X-ray diffractometer (XRD, D/Max-2400). The XRD patterns of ZnO nanosheets are shown in Figure 1A. All the diffraction peaks of the samples were well-indexed to the fibrous zincite structure of ZnO, and no other diffraction peaks corresponded to impurities, and the results indicated that the final product is a higher purity form of ZnO. Figure 1B shows the corresponding EDX pattern of ZnO nanosheets, further confirming that the higher purity ZnO consists of Zn and O (the presence of Cu and C signals came from the carbon-coated Cu grids used in the TEM measurements).

**FIGURE 2:**
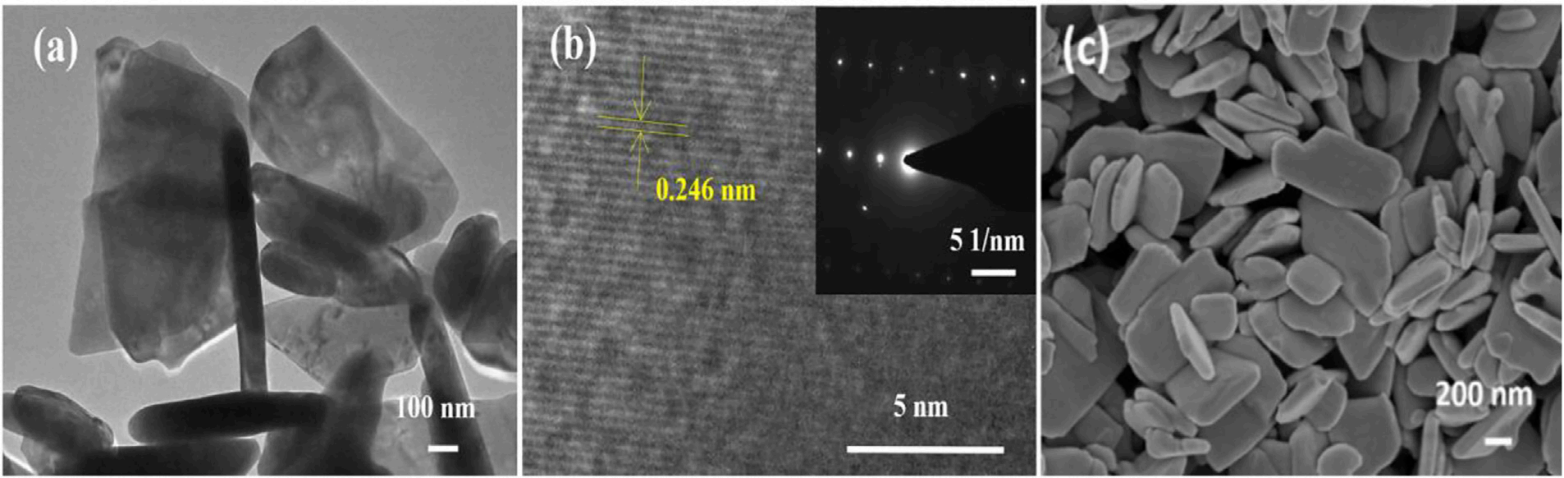
TEM, HRTEM, and SEM images of ZnO nanomaterials.**(A)**TEM images of ZnO nanosheets; **(B)** HRTEM images of ZnO nanosheets; and **(C)** SEM images of ZnO nanosheets.

The morphological analysis of the prepared ZnO products was carried out by FESEM and TEM. Figure 2C shows the FESEM image of the nanosheets, which form an irregular structure with a length of about 200–900 nm and a thickness of about 600 nm.

The morphological characteristics of ZnO nanosheets were further confirmed by TEM images (Figure 2A). The HRTEM image of the material is shown in Figure 2B, and the inset of Figure 2B shows its selected area electron diffraction (SAED) pattern. The lattice stripe spacing in the HRTEM image of the ZnO nanosheet is measured to be 0.246 nm, which corresponds to the (101) crystal plane of the fibrous ZnO structure, and the corresponding selected area electron diffraction (SAED) pattern is a symmetric point lattice, indicating that the ZnO nanosheet is a single-crystal structure.

### 3.2 Cell morphology observation

**FIGURE 3:**
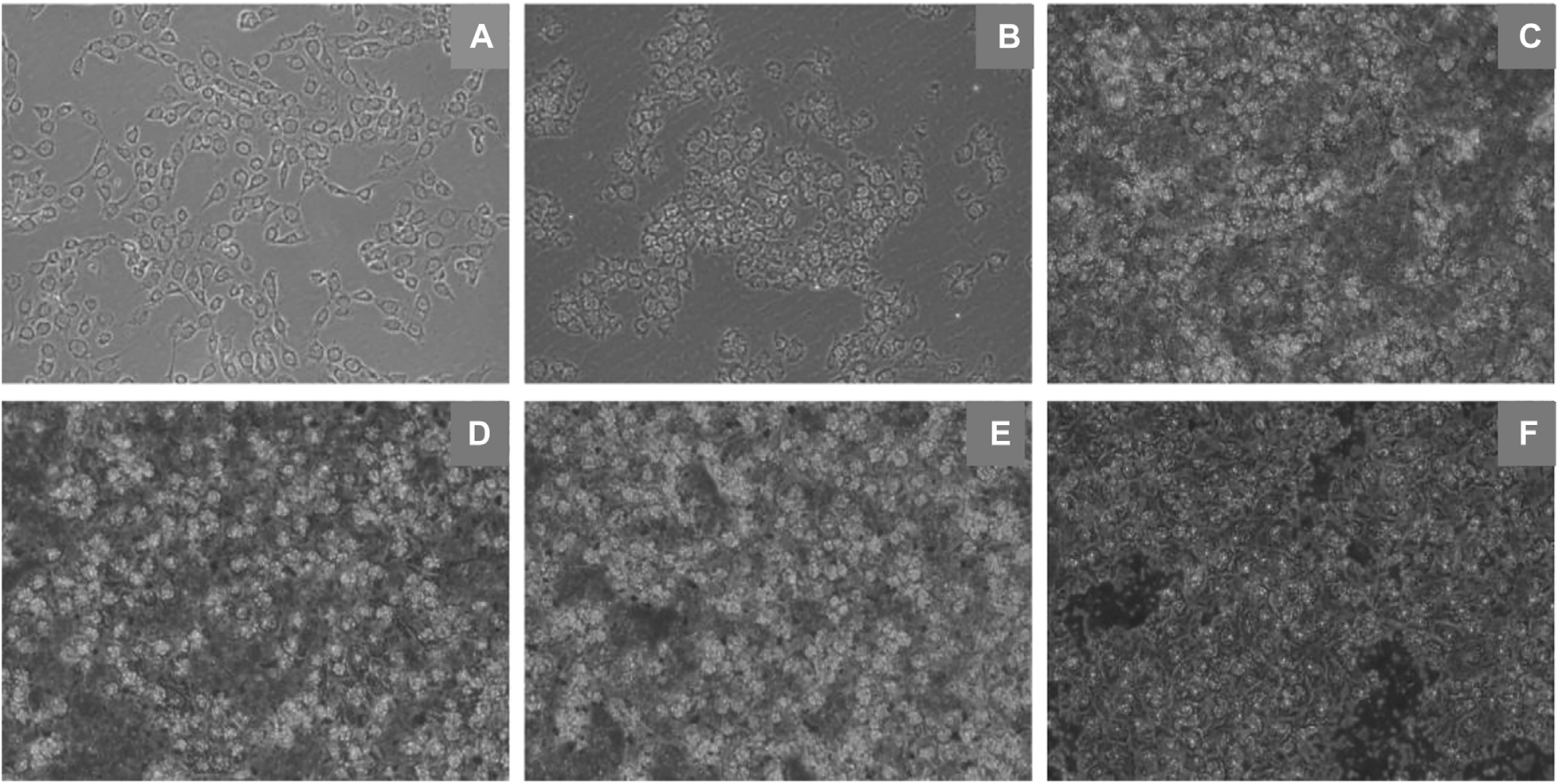
Morphological changes of EBL cells induced by ZnO nanosheets (×10 inverted fluorescence microscope). **(A)** Control; **(B–F)** Changes in morphology of cells in different concentrations (12.5 μg ml^−1^, 25 μg ml^−1^, 50 μg ml^−1^, 100 μg ml^−1^, and 200 μg ml^−1^) of flaky ZnO nanomaterial.

An inverted microscope was used to observe cell morphological changes. EBL cells were exposed to DMEM with different concentrations of ZnO nanosheets. After 24 h of incubation, the cells in the control group were observed to grow against the wall in a polygonal shape with a relatively neat arrangement, completed morphology, and uniform size under the microscope. Compared with the control group, there was membrane deformation in the treated group, the cells were severely vacuolated and poorly adhered to the wall, and there were more suspended cells (Figure 3).

Studies have reported that ZnO nanomaterials can enter A549 cells through endocytosis and release Zn^2+^ with the help of lysosomes, resulting in the increasing concentration of intracellular Zn^2+^ (Wu et al., 2019b). They further confirmed this process through TEM analysis. Obvious vacuoles were observed in cells treated with ZnO nanomaterials, and some vacuoles contained black spots, namely, nano-ZnO. The vesicles produced during the endocytosis of nano-ZnO may contribute to the formation of endosomes.

### 3.3 Effect of different concentrations of ZnO nanosheets on cell viability

The effects of different concentrations of ZnO nanosheets on the cell viability of EBL cells after 24, 48, and 72 h were examined using the MTT kit. The results showed that at low doses, the cell viability of the treatment group was significantly lower than that of the control group, especially at the treatment dose of 25 μg ml^−1^; there was a significant difference within the group (*p* < 0.05), and the cell viability increased with the increase of ZnO nanosheet concentration (*p* < 0.05) (Table 1).

**TABLE 1:** Viability of EBL cells incubated with ZnO nanosheets at different periods (*n* = 3,x ± S).

Concentration (μg^.^ml^−1^)	Cell viability (%)		
	24 h	48 h	72 h
0	100	100	100
12.5	5.64 ± 0.70^a^	3.37 ± 0.54^a^	2.00 ± 0.15^a^
25	0.80 ± 5.83^a^	0.74 ± 0.16^a^	0.65 ± 0.21^a^
50	2.74 ± 0.39^a^	2.09 ± 0.07^a^	2.00 ± 0.28^a^
100	7.64 ± 0.08^abc^	7.42 ± 0.13^abc^	7.04 ± 0.16^abc^
200	29.89 ± 2.99^abcd^	18.83 ± 2.81^abcd^	14.36 ± 5.31^aabcd^

^a^ *p* < 0.05 vs. 0 μg ml^−1^; a: *p* < 0.05 vs. 12.5 μg ml^−1^; b: *p* < 0.05 vs. 25 μg ml^−1^; c: *p* < 0.05 vs. 50 μg ml^−1^; and d: *p* < 0.05 vs. 100 μg ml^−1^.

**FIGURE 4:**
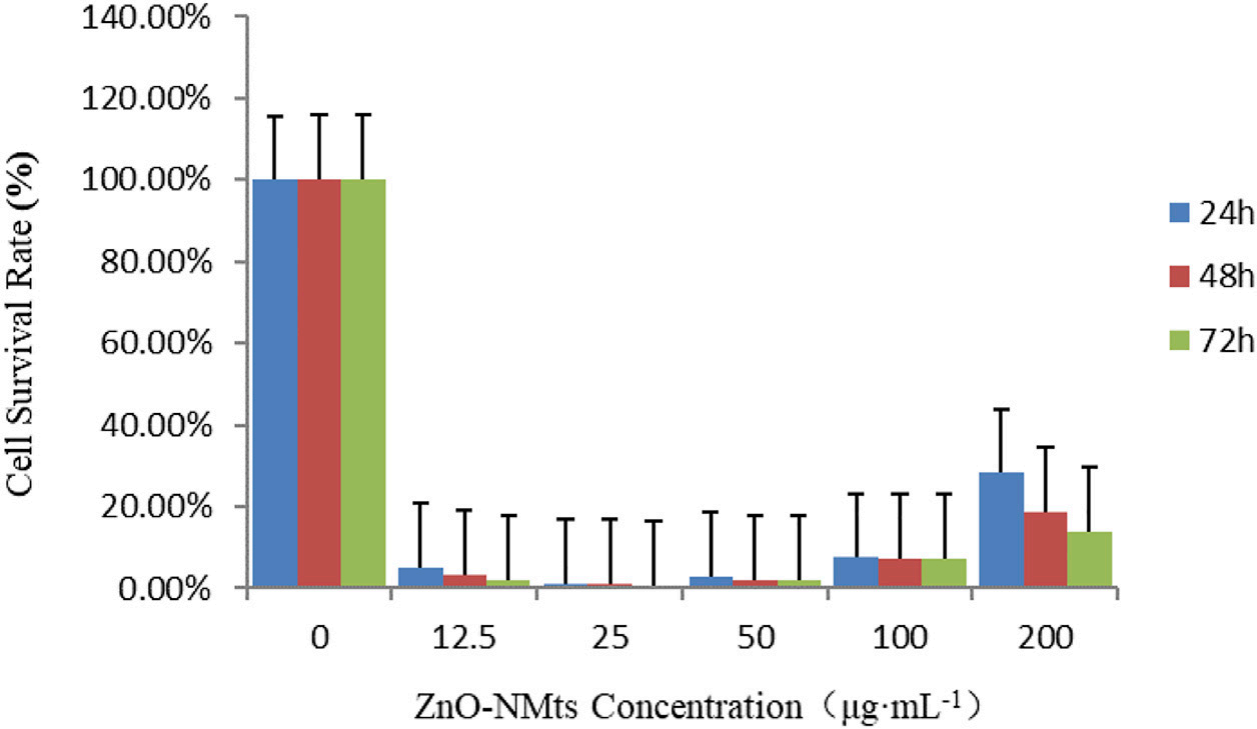
Viability of EBL cells treated with ZnO nanomaterials for 24, 48, and 72 h. As shown in the figure, the survival rate of EBL cells decreased with the increase in the action time of ZnO nanomaterials.

The results showed that after using the MTT kit to treat EBL cells at different times, compared with the control group, the cell viability of the treatment group decreased significantly, and with the increase in action time, the cell viability decreased. The results showed that the viability of EBL cells decreased with the increase in action time of ZnO nanomaterials (Figure 4).

### 3.4 Effect of ZnO nanosheets on apoptosis of EBL cells

#### 3.4.1 Effect of different concentrations of ZnO nanosheets on cellular caspase-3 activity

**FIGURE 5:**
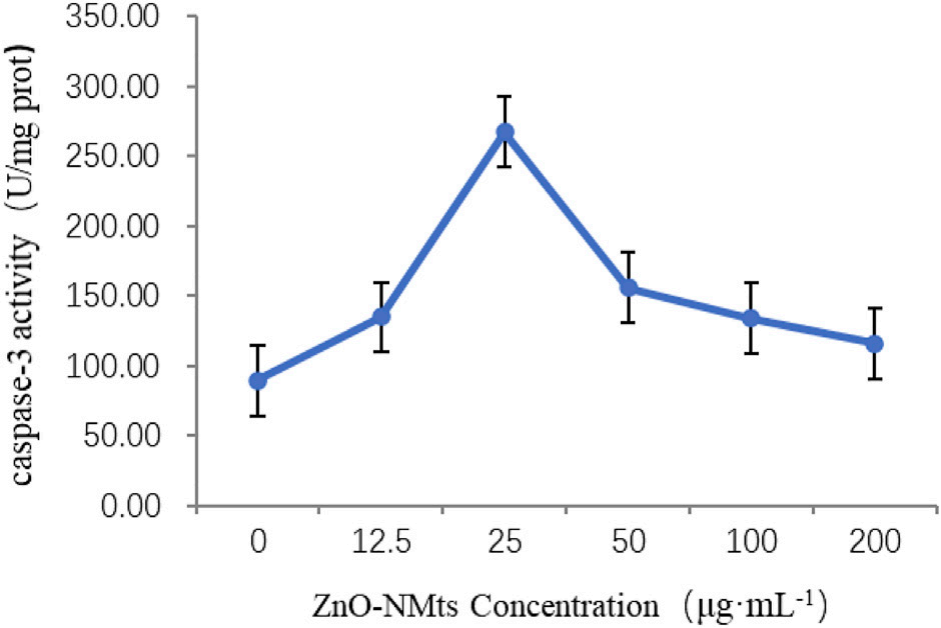
Effect of ZnO nanosheets on the activity of caspase-3 in cells. As shown in the figure, the highest caspase-3 activity was observed when the nanomaterial concentration was 25 μg ml^−1^ and then decreased with the increasing concentration.

Caspase-3 is the dominant player in the apoptotic cascade response and is the common pathway for all apoptotic signals. Caspase-3 was used to detect the effect of ZnO nanosheets on the caspase-3 activity of EBL cells at different concentrations of ZnO nanosheets. The results showed that caspase-3 activity increased with the increasing concentration at low concentrations, and the highest caspase-3 activity was observed when the nanomaterial concentration was 25 μg ml^−1^ and then decreased with the increasing concentration. The optimal concentration of ZnO nanosheets to inhibit cell growth was 25 μg ml^−1^ (Figure 5).

#### 3.4.2 Effect of ZnO nanosheets on the caspase-3 activity of cells at different action times

**FIGURE 6:**
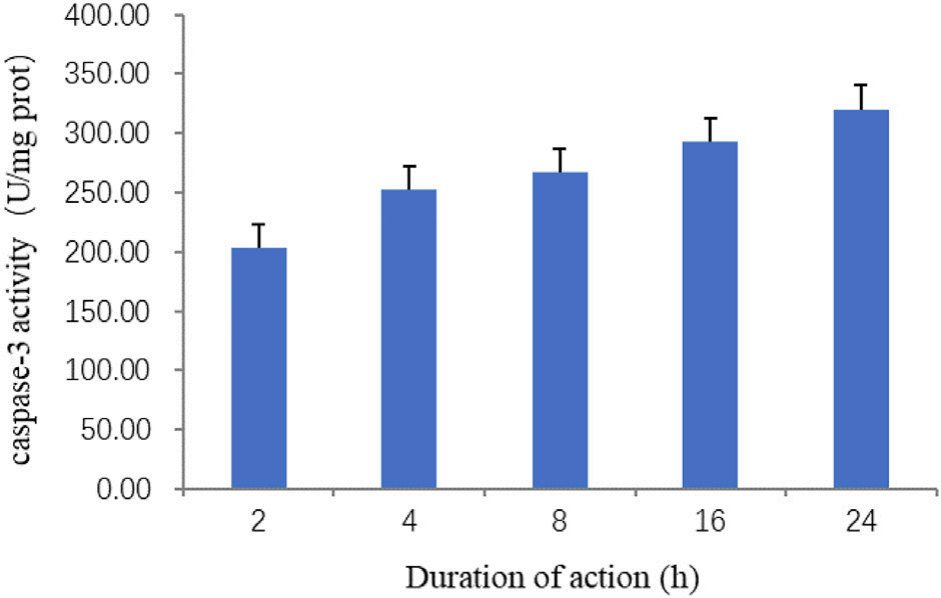
Effect of ZnO nanosheets at different action times on the activity of caspase-3 in cells. As shown in the figure, caspase-3 activity increased with the increase in the time of the action of ZnO nanosheets (200 μg ml)^−1^ on the cells, and the highest -3 activity was reached at 24 h.

A caspase-3 activity detection kit was used to detect the changes in caspase-3 activity of the ZnO nanosheets on EBL cells at 0, 2, 4, 8, 16, and 24 h, respectively. The results showed that caspase-3 activity increased with the increase in the time of the action of ZnO nanosheets (200 μg ml^−1^) on the cells, and the highest caspase-3 activity was reached at 24 h. The results indicated that the apoptosis of EBL cells was more severe with the increase in the action time of ZnO nanomaterials (Figure 6). The present experiment confirmed that ZnO nanosheets induced EBL cells to activate caspase-3 and promote apoptosis.

#### 3.4.3 Apoptosis detection by flow cytometry

**FIGURE 7:**
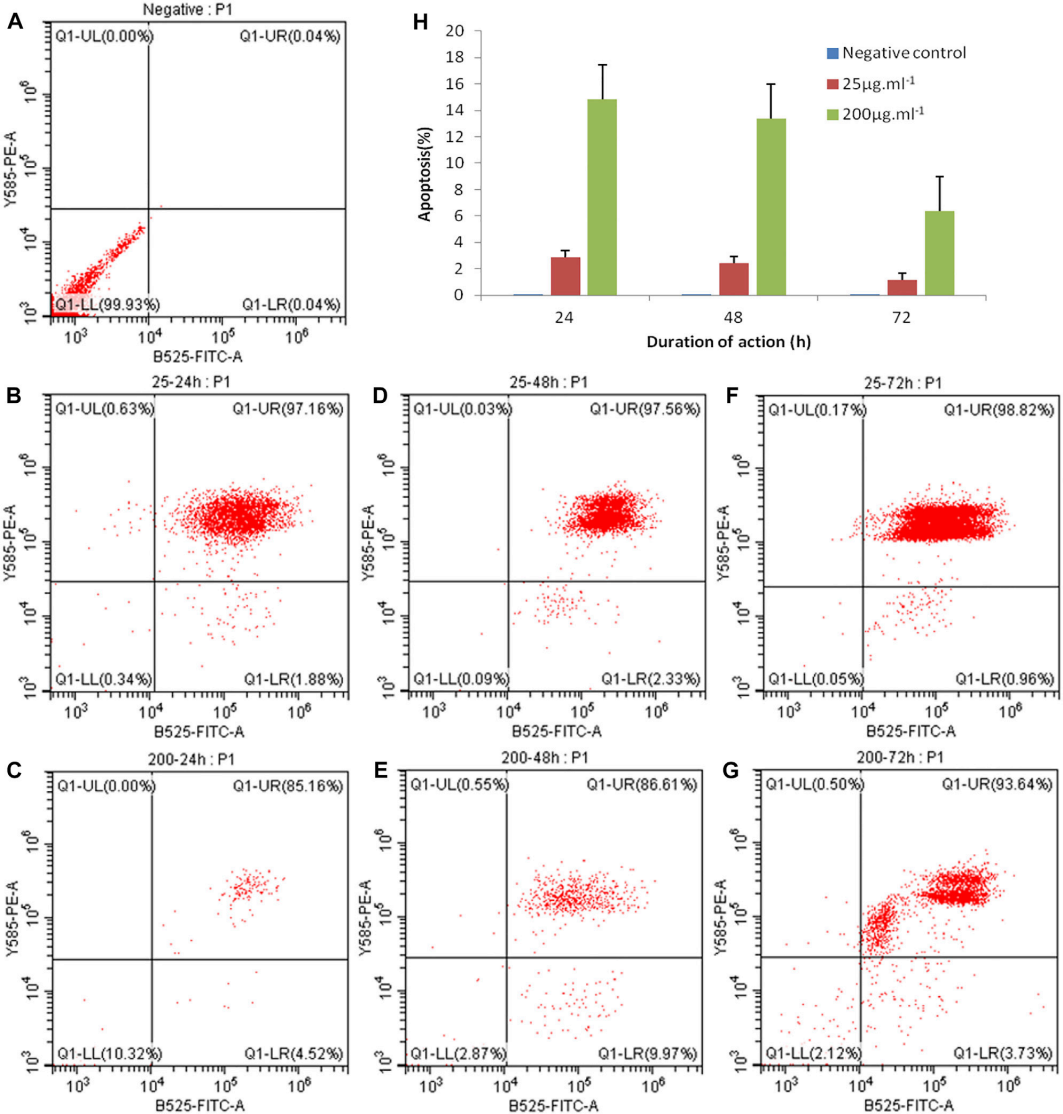
EBL cell apoptosis caused by ZnO nanosheet detection using FCM. **(A)** Negative control; **(B)** 25 μg mL^−1^–24 h; **(C)** 200 μg ml^−1^–24 h; **(D)** 25 μg mL^−1^–48 h; **(E)** 200 μg ml^−1^–48h; **(F)** 25 μg ml^−1^–72 h; and **(G)** 200 μg ml^−1^–72 h. As shown in the figure, the concentrations of 25 μg ml^−1^ and 200 μg ml^−1^ of ZnO nanosheets were applied to EBL cells for 24, 48, and 72 h, respectively, and severe apoptosis was observed in each treatment group, which was significantly different from the control group.

Apoptosis, also known as programmed cell death (PCD), is the process of active termination of life under the regulation of apoptosis-related genes, which is involved in the activation, expression, and regulation of cell-related genes, an orderly process that maintains the homeostasis and stability of the internal environment (Wu et al., 2019c). FITC-annexin V/PI dual fluorescent labeling was used, and PI was employed to detect apoptosis. The results showed that the concentrations of 25 μg ml^−1^ and 200 μg ml^−1^ of ZnO nanosheets were applied to EBL cells for 24, 48, and 72 h, respectively, and severe apoptosis was observed in each treatment group, which was significantly different from the control group (Figure 7).

### 3.5 Effect of ZnO nanosheets on intracellular reactive oxygen species (ROS) in EBL cells

**FIGURE 8:**
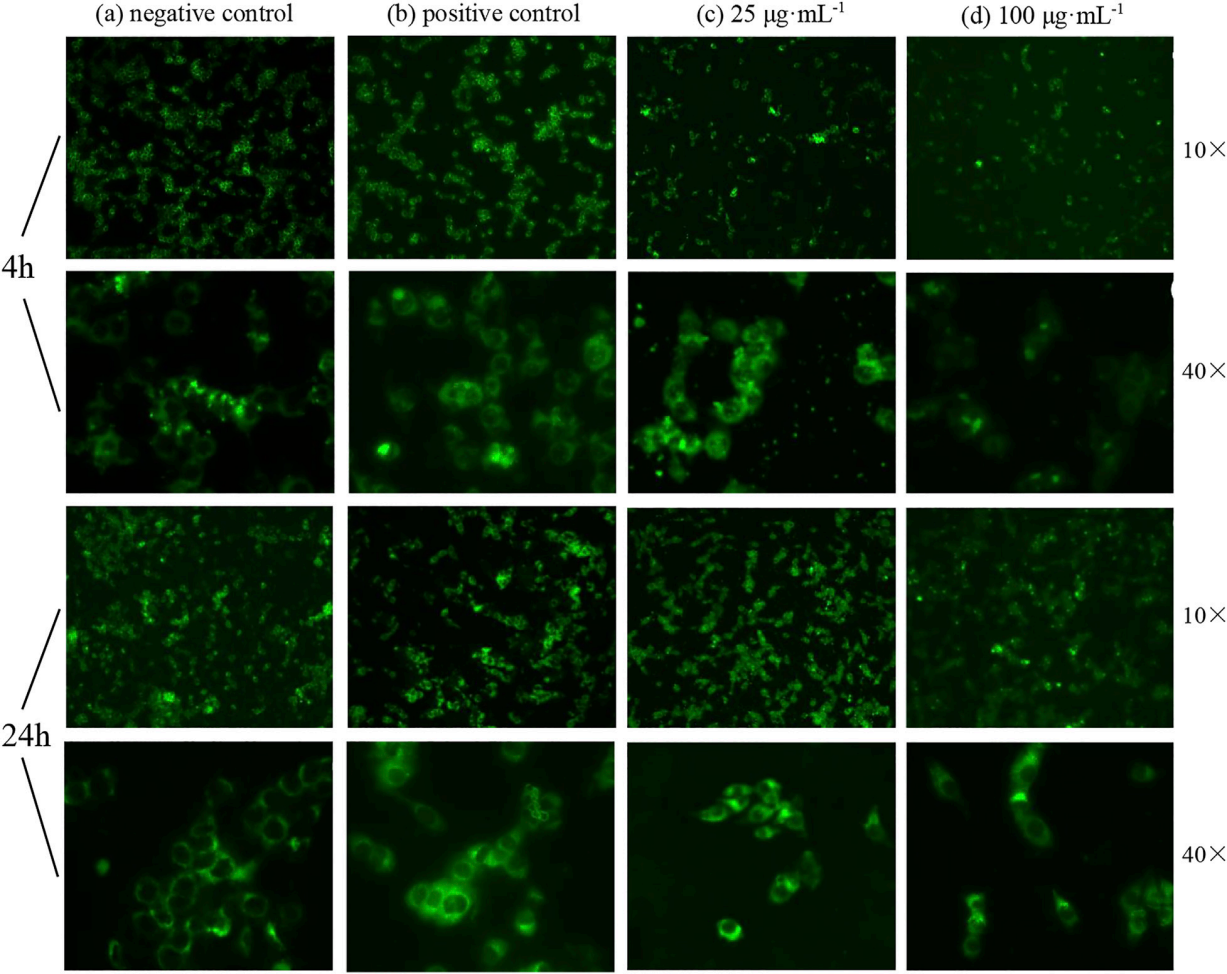
Fluorescence changes of cells treated with ZnO nanosheets for different hours (using ×10 or ×40 inverted fluorescence microscopes). **(A)** Negative control; **(B)** positive control; **(C)** concentration of 25 μg ml^−1^; **(D)** concentration of 100 μg ml^−1^. As shown in the figure, the intracellular ROS level increased significantly with the increase in nanomaterial action time on cells; different degrees of welling or deformation were observed in some cells under high magnification, and green fluorescence showed uneven and irregular cell changes.

Oxidative damage of cells may also lead to apoptosis. Apoptosis was detected using ROS levels, and the results showed that a very bright green fluorescence was observed from the positive control group under inverted fluorescence microscopy; the fluorescence from the negative control group was faint. After the effect of ZnO nanosheets at concentrations of 25 μg ml^−1^ and 100 μg ml^−1^ on EBL cells for 4 h, the green fluorescence was observed under ×10 and ×40 inverted fluorescence microscopes showing different degrees of enhancement, and the fluorescence of the treated group was stronger than that of the negative control group and weaker relative to the positive control group compared with the control group and the fluorescence at the concentration of 25 μg ml^−1^. The fluorescence was brighter at 25 μg ml^−1^ than that at 100 μg ml^−1^. The fluorescence intensity of both control and treated groups was significantly enhanced after 24 h of action (Figure 8).

The results showed that the intracellular ROS level increased significantly with the increase in nanomaterial action time on cells; different degrees of welling or deformation were observed in some cells under high magnification, and green fluorescence showed uneven and irregular cell changes.

Nanomaterials are now widely used in the biomedical field because of their unique structure and properties. In recent years, the toxic and immunological effects of nanomaterials on cells have also become hot topics of research studies (Yuan et al., 2019). Some researchers believe that the generation of intracellular reactive oxygen species (ROS) is due to the cytotoxicity of nanomaterials (Mao and Cai, 2021). Some studies have shown that nanomaterials interact with cell membranes to generate large amounts of ROS, which causes structural damage to the cell membranes and leads to the imbalance of the intracellular environment, resulting in cell apoptosis (Król et al., 2017). Karkossa et al. (2021) found that the production of ROS and oxidative stress responses are the main ways in which nanomaterials cause a variety of biological effects.

In this study, the effects of ZnO nanosheets on the morphology of fetal bovine lung epithelial cells and the effects of cytotoxicity, intracellular reactive oxygen levels, and apoptosis were investigated in detail. Combined with the observation of cell morphology and the detection of apoptosis markers, this study fully proved that ZnO nanoparticles can inhibit cell proliferation and promote cell apoptosis. It was found for the first time that the cell viability increased with the increase in material concentration. This result needs to be further verified, and its mechanism needs to be studied to reveal the principle, which will be helpful for the application of nanomaterials in immortalized cells. Studies have confirmed that EBL cells induced by ZnO nanomaterials can activate caspase-3 and promote apoptosis. At the same time, 25 μg ml^−1^ concentration can also be used as a reference concentration to directly act on tumor cells, opening a new window for the application of nanomaterials in inhibiting tumor cells. These studies can further clarify the toxic effects of ZnO nanomaterials on cells. The research study on the biological effects of nanomaterials has just begun. In further research, it is very important to clarify ideas and provide the basis for the design of nanomaterials with different biological effects.

## Data Availability

The original contributions presented in the study are included in the article/Supplementary material; further inquiries can be directed to the corresponding authors.

## Glossary

DCFH-DA: 2′,7′-dichlorodihydrofluorescein
DMEM: Dulbecco’s modified Eagle’s medium
DMSO: dimethyl sulfoxide
EBL cells: embryonic bovine lung cells
FBS: fetal bovine serum
MTT: methyl thiazolyl tetrazolium
OD: optical density
PBS: phosphate-balanced solution
ROS: reactive oxygen species
SEM: scanning electron microscope
TEM: transmission electron microscope
